# Porous molybdenum carbide nano-octahedrons synthesized via confined carburization in metal-organic frameworks for efficient hydrogen production

**DOI:** 10.1038/ncomms7512

**Published:** 2015-03-11

**Authors:** Hao Bin Wu, Bao Yu Xia, Le Yu, Xin-Yao Yu, Xiong Wen (David) Lou

**Affiliations:** 1 School of Chemical and Biomedical Engineering, Nanyang Technological University, 62 Nanyang Drive, Singapore 637459, Singapore

## Abstract

Electrochemical water splitting has been considered as a promising approach to produce clean and sustainable hydrogen fuel. However, the lack of high-performance and low-cost electrocatalysts for hydrogen evolution reaction hinders the large-scale application. As a new class of porous materials with tunable structure and composition, metal-organic frameworks have been considered as promising candidates to synthesize various functional materials. Here we demonstrate a metal-organic frameworks-assisted strategy for synthesizing nanostructured transition metal carbides based on the confined carburization in metal-organic frameworks matrix. Starting from a compound consisting of copper-based metal-organic frameworks host and molybdenum-based polyoxometalates guest, mesoporous molybdenum carbide nano-octahedrons composed of ultrafine nanocrystallites are successfully prepared as a proof of concept, which exhibit remarkable electrocatalytic performance for hydrogen production from both acidic and basic solutions. The present study provides some guidelines for the design and synthesis of nanostructured electrocatalysts.

## Abstract (web summary)

There is extensive research into non-platinum electrocatalysts for hydrogen evolution. Here, the authors report a molybdenum carbide catalyst, prepared via the carburization of a copper metal-organic framework host/molybdenum-based polyoxometalates guest system, and demonstrate its catalytic activity.

## Graphical abstract

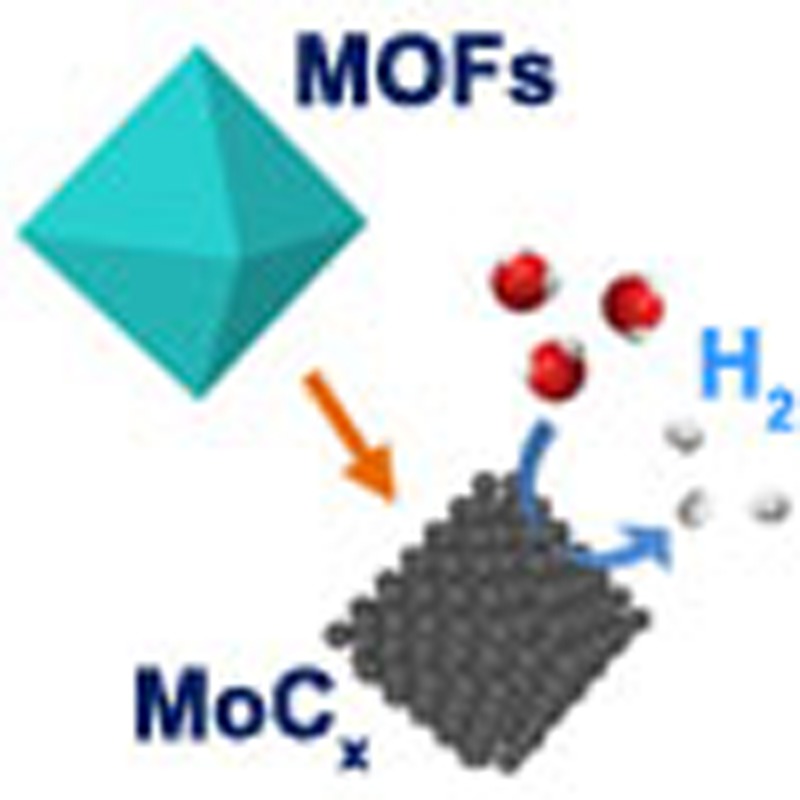

The rapid growth of global energy consumption and the associated environmental issues have triggered the urgent demand for renewable and clean energy sources. Electrochemical water splitting driven by solar energy has been considered as an attractive approach to produce hydrogen (H_2_) fuel, a sustainable, secure and environmentally benign energy vector^1^,^2^,^3^. Efficient water splitting requires high-performance electrocatalysts to promote the hydrogen evolution reaction (HER). Platinum (Pt) has been identified as the most active HER catalyst, whereas its high cost and low abundance hinder the large-scale application^4^. Therefore, numerous efforts have been devoted to search for noble metal-free HER catalysts^5^,^6^,^7^,^8^,^9^,^10^,^11^,^12^,^13^,^14^,^15^,^16^,^17^,^18^. Transition metal carbides, such as molybdenum (Mo) and tungsten (W) carbides have been under investigation for decades in the fields of catalysis in view of their high similarity to Pt-group metals^19^,^20^, and have been recently suggested as promising electrocatalysts for HER^21^,^22^. Particularly, β-phase molybdenum carbide (β-Mo_2_C) has been demonstrated as a highly active HER catalyst even as bulky particles^23^, and the performance could be further improved by constructing proper nanostructures^24^,^25^,^26^,^27^. However, controllable synthesis of nanostructured metal carbides with small nanocrystallites and desirable porosity towards high electrocatalytic activity still remains as a great challenge, due to the difficulty to achieve uniform carburization and the inevitable coalescence of nanoparticles at high reaction temperature.

In recent years, metal-organic frameworks (MOFs) have emerged as a new class of porous materials with widespread applications in gas storage/separation, catalysis, sensing and drug delivery^28^,^29^,^30^. Moreover, syntheses of functional materials from MOFs have drawn fast-growing interests as well. The periodically porous and hybrid structure of MOFs offer unique benefits for the fabrication of carbon and/or metal-based nanostructured materials. Specifically, MOFs-derived porous carbon, metal oxides, metal/carbon and metal oxide/carbon nanocomposites have been reported by using MOFs as both the precursor and template^31^,^32^,^33^,^34^,^35^,^36^. For example, MOFs-derived nanoporous carbon materials exhibit exceptionally high surface area and uniform porosity, which are largely originated from the ordered and porous structure of MOFs^32^,^33^,^37^. We have previously used Prussian blue cubic microcrystals to prepare various iron oxide-based hollow microboxes with complex shell structures and compositions^38^,^39^. This category of MOFs-derived materials has been recently extended to iron carbide^40^. However, in most of these studies, MOFs are exclusively used as the sole precursor. Although the huge family of MOFs has covered a wide range of metal species, these conventional MOFs-derived strategies are mainly based on a limited number of MOFs that are easily obtainable and/or with controllable morphology. Consequently, the reported MOFs-derived metal-based materials are typically limited to a handful of elements (for example, Zn, Cu, Co, Fe and so on).

In this work, we develop a MOFs-assisted strategy for synthesizing porous molybdenum carbide octahedral nanoparticles (denoted as MoC_*x*_ nano-octahedrons) that consist of very small nanocrystallites as electrocatalysts for efficient hydrogen production. Distinct from previous studies, the present synthesis strategy relies on the *in situ* and confined carburization reaction between the organic ligands (or their derived carbon-based species) of MOFs and guest polyoxometalates (POMs) that reside in the pores of the MOFs host. The introduction of guest metal species into the MOFs host as co-precursor enables easy synthesis of early transition metal (for example, Mo, W and V) carbides, which are difficult to obtain from a single MOFs source. Meanwhile, these non-coordinating POMs are also uniformly distributed and surrounded by organic ligands in atomic scale, thus guaranteeing *in situ* and homogeneous carburization reaction that produces small carbide nanocrystallites. In addition, the carburization process would be confined within the carbonaceous matrix derived from organic ligands of MOFs, which effectively prevents the agglomeration and coalescence of *in situ*-generated carbide nanocrystallites. As a proof of concept, we demonstrate the synthesis of molybdenum carbide using this MOFs-assisted approach in view of its promising application in catalysis (for example, for HER) and the easy encapsulation of Mo-based POMs in a particular MOFs host as discussed shortly. Interestingly, the as-prepared molybdenum carbide is in a *η*-MoC phase, which is unexpected at a relatively low carburization temperature of 800 °C and has not been well investigated for electrocatalytic hydrogen production^41^. Benefiting from the porous and robust structure, as well as the ultrafine primary nanocrystallites, the as-prepared molybdenum carbide nano-octahedrons exhibit remarkable electrocatalytic activity for HER in both acidic and basic conditions. The present strategy is also applicable to synthesize W and Mo-W carbides, and could be extended to other early transition metals as well.

## Results

### MOFs-assisted synthesis strategy for molybdenum carbide

**Figure 1:**
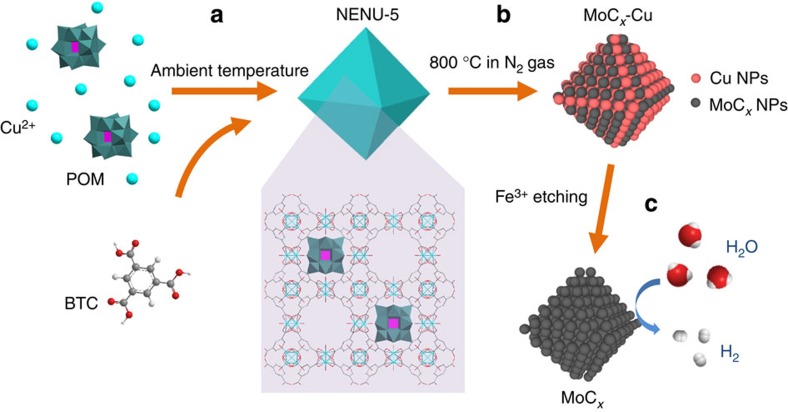
Schematic illustration of the synthesis procedure for porous MoC_*x*_ nano-octahedrons. (**a**) Synthesis of NENU-5 nano-octahedrons with Mo-based POMs residing in the pores of HKUST-1 host. (**b**) Formation of MoC_*x*_-Cu nano-octahedrons after annealing at 800 °C. (**c**) Removal of metallic Cu nanoparticles by Fe^3+^ etching to produce porous MoC_*x*_ nano-octahedrons for electrocatalytic hydrogen production.

The overall synthesis route to prepare porous MoC_*x*_ octahedral sub-micrometre-sized particles (denoted as MoC_*x*_ nano-octahedrons) as efficient HER catalysts is illustrated in Fig. 1. We choose a unique MOFs-based compound as the precursor with a formula of [Cu_2_(BTC)_4/3_(H_2_O)_2_]_6_[H_3_PMo_12_O_40_] (NENU-5; BTC=benzene-1,3,5-tricarboxylate), which is based on a well-studied Cu-based MOF [HKUST-1; Cu_3_(BTC)_2_(H_2_O)_3_] with Mo-based Keggin-type POMs (H_3_PMo_12_O_40_) periodically occupying the largest pores^42^. In this work, NENU-5 nano-octahedrons are synthesized by a facile and scalable co-precipitation method at ambient temperature. The as-prepared NENU-5 nano-octahedrons containing substantial amount of Mo are directly heated at 800 °C in N_2_ gas flow to produce MoC_*x*_-Cu. During the annealing process, the Mo-based POMs react with carbonaceous species derived from BTC ligands to form MoC_*x*_ nanocrystallites, meanwhile Cu^2+^ clusters are reduced to metallic Cu. Finally, MoC_*x*_ nano-octahedrons composed of small nanocrytallites are obtained by etching the metallic Cu nanoparticles with aqueous solution of FeCl_3_ (2 Fe^3+^+Cu→Cu^2+^+2 Fe^2+^), and used as electrocatalysts for HER.

### Synthesis of porous MoC_*x*_ nano-octahedrons

**Figure 2:**
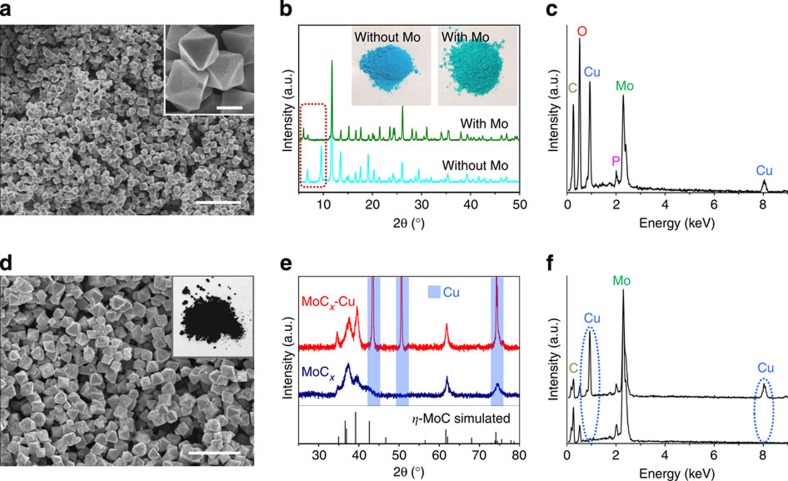
Characterizations of precursors and MoC_*x*_ nano-octahedrons. (**a**) FESEM image (inset: magnified image; scale bar, 500 nm) of the as-prepared NENU-5 nano-octahedrons; scale bar, 5 μm. (**b**) XRD patterns (inset: digital photos) of NENU-5 (with Mo) and HKUST-1 (without Mo). (**c**) EDX spectrum of the as-prepared NENU-5 nano-octahedrons. (**d**) FESEM image (inset: digital photo) of porous MoC_*x*_ nano-octahedrons; scale bar, 2 μm. (**e**) XRD patterns and (**f**) EDX spectrums of MoC_*x*_-Cu and MoC_*x*_ nano-octahedrons.

Figure 2a shows typical field-emission scanning electron microscopy (FESEM) images of the as-prepared NENU-5 particles in octahedral or slightly truncated octahedral shape with a sub-micrometre size of ~800 nm. The size of the as-prepared NENU-5 particles can be easily tuned by varying the addition amount of L-glutamic acid that slows down the nucleation rate (Supplementary Fig. 1). Powder X-ray diffraction (XRD) pattern shown in Fig. 2b (upper curve) confirms the phase purity as well as the excellent crystallinity of the NENU-5 nano-octahedrons. The XRD pattern of HKUST-1 is also provided for comparison (lower curve in Fig. 2b), which exhibits different diffraction peaks especially below 10°. Moreover, the successful incorporation of Mo-based POMs in the HKUST-1 framework can be visually verified by the green colour of the product, distinct from the blue colour of pristine HKUST-1 (insets of Fig. 2b)^43^. The chemical composition is further examined by energy-dispersive X-ray spectroscopy (EDX). The spectrum (Fig. 2c) evidently shows the presence of substantial amount of Mo in the as-prepared NENU-5 nano-octahedrons. Moreover, a Mo/Cu atomic ratio of ~0.7 suggested by EDX quantitative analysis is lower than the theoretical value of 1 in NENU-5. Therefore, some vacancies of POMs exist in the as-prepared NENU-5 nano-octahedrons, which are probably due to the low synthesis temperature and/or the blocking of pores by other species from the solution. Such vacancies are also responsible for the low intensity of characteristic XRD peaks in small angle region^43^. Moreover, the vacancies of POMs would reduce the yield of MoC_*x*_ and possibly result in less uniform distribution of MoC_*x*_ nanocrystallites, which should be minimized in future studies.

After the annealing and etching processes, the MoC_*x*_ sample is obtained as a black powder (inset of Fig. 2d). FESEM image (Fig. 2d) reveals that the octahedral shape of the particles is well retained, while the surface becomes slightly rougher. The complete removal of Cu particles by Fe^3+^ etching is confirmed by both XRD and EDX analyses. As shown in Fig. 2e, the three strong and sharp peaks from metallic Cu in the XRD pattern of MoC_*x*_-Cu sample (upper curve) are no longer observed in the pattern of MoC_*x*_ sample (lower curve). Moreover, EDX spectrum of the MoC_*x*_ sample confirms the main composition of Mo and C, and also excludes the presence of Cu (Fig. 2f). Surprisingly, the XRD pattern of MoC_*x*_ sample shows distinct results from previous reports that typically produce β-Mo_2_C by annealing mixtures of molybdenum salts and organic compounds at similar temperatures^25^,^26^,^27^,^44^. The pattern can be satisfactorily assigned to hexagonal *η*-MoC phase (Supplementary Fig. 2) that is usually produced at much higher temperatures^45^,^46^ or in the presence of NiI_2_ with short reaction duration^41^. In our system, the *η*-MoC phase can be produced between 750 and 850 °C with little alteration in the XRD patterns (Supplementary Fig. 3). This unusual result implies the distinct characteristics of chemical reactions confined in the MOFs matrix, which is also exemplified by the uncommon synthesis of pure brookite-phase TiO_2_ through replication of MOFs^47^. Considering the likelihood to form substoichiometric *η*-MoC_1−*x*_ phases^46^, and the difficulty to determine the exact chemical composition due to the presence of extra amorphous carbon (as discussed shortly), the as-prepared molybdenum carbide is denoted as MoC_*x*_ in this work. Moreover, the diffraction peaks are significantly broadened, suggesting the very small size of nanocrystallites due to the effective inhibition of coalescence and crystal growth during the confined carburization process. Meanwhile, excessive growth of Cu particles in the MoC_*x*_-Cu sample as suggested by the XRD pattern (see upper curve in Fig. 2e) still occurs during the annealing process, which is probably related to the relatively low melting point of Cu.

### Structural characterizations of MoC_*x*_ nano-octahedrons

**Figure 3:**
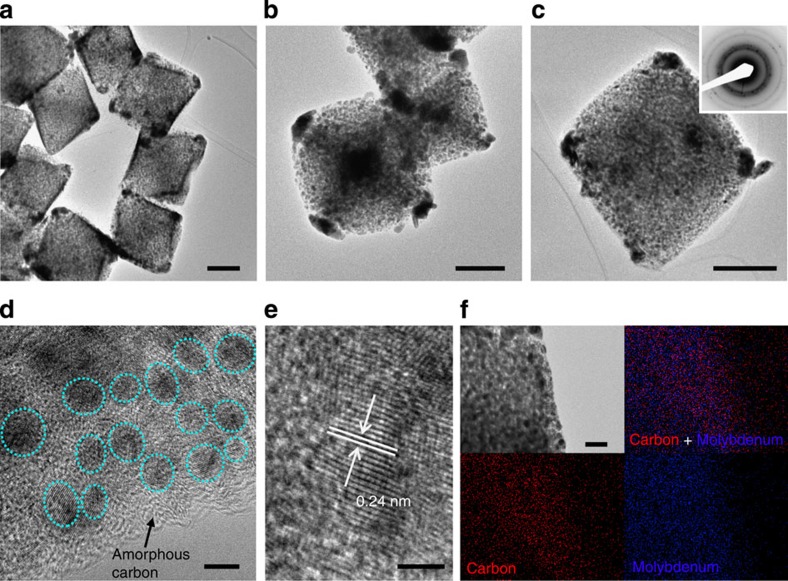
Characterizations of MoC_*x*_ nano-octahedrons. (**a**–**c**) TEM images (scale bar, 200 nm; inset of c: SAED pattern), (**d**) magnified TEM image (scale bar, 5 nm), (**e**) HRTEM image (scale bar, 2 nm) and (**f**) elemental mapping (red: carbon; blue: molybdenum; scale bar, 50 nm) of porous MoC_*x*_ nano-octahedrons.

The structure of the as-prepared MoC_*x*_ nano-octahedrons is further examined by transmission electron microscopy (TEM) as depicted in Fig. 3. The sample appears as rhombic or cubic particles under TEM observation as the projections of octahedral particles from different directions (Fig. 3a–c). A closer examination on the MoC_*x*_ nano-octahedrons reveals the highly porous texture throughout the whole particle. Each MoC_*x*_ nano-octahedron is composed of numerous small nanocrystallites, and the polycrystalline nature is confirmed by selected-area electron diffraction (SAED) pattern as shown in the inset of Fig. 3c. An interesting observation is that some large particles appear at the corners of the octahedral particles. This is probably due to the higher surface activity and stress in these regions that cause easy collapse of the MOFs matrix and subsequent growth of MoC_*x*_ nanocrystallites during the high-temperature reaction. Indeed, our initial attempt to prepare MoC_*x*_ using much smaller NENU-5 nanoparticles (shown in Supplementary Fig. 1a) with high surface activity results in strongly aggregated particles with a poorly crystallized β-Mo_2_C phase that cannot be well dispersed into suspension for subsequent electrochemical measurements (Supplementary Fig. 4). Therefore, a robust secondary structure with a moderate size (for example, sub-micrometre-sized NENU-5 particles) is essential to successfully carry out the confined carburization reaction while providing large exposed surface for catalytic purpose.

A closer TEM examination on the edge of a MoC_*x*_ nano-octahedron gives more details of the nanostructure. Figure 3d clearly shows that numerous MoC_*x*_ clusters (darker area with visible lattice fringes indicated by green circles) are embedded in amorphous carbon matrix. Judging from their crystal lattices, the size of primary MoC_*x*_ nanocrystallites is typically within 5 nm, although some seem to slightly aggregate and appear as larger clusters. The presence of amorphous carbon is also verified by thermogravimetric analysis and Raman spectrum (Supplementary Fig. 5), which might play important roles in prohibiting the growth of MoC_*x*_ nanocrystallites and stabilizing the octahedral particles. A representative high-resolution (HR) TEM image (Fig. 3e) clearly shows lattice fringes with an interplanar distance of 0.24 nm, corresponding to the (006) planes of *η*-MoC. The uniform distribution of Mo and C elements is illustrated by EDX elemental mappings shown in Fig. 3f. Moreover, the MoC_*x*_ nano-octahedrons exhibit a highly mesoporous structure, as evidenced by a high specific Brunauer–Emmett–Teller surface area of 147 m^2^ g^−1^ and abundant mesopores mainly distributed in the range of 4–10 nm (Supplementary Fig. 6). The mesopores in the MoC_*x*_ nano-octahedrons are obviously larger than the pores in the pristine HKUST-1, which would be related to the substantial mass loss (that is, loss of C, H, O, Cu elements) during the carburization process. Such a porous structure with high uniformity is largely inherited from the ordered porous architecture of the MOFs precursor, which would benefit the application in electrocatalysis.

### Electrocatalytic performance for HER

**Figure 4:**
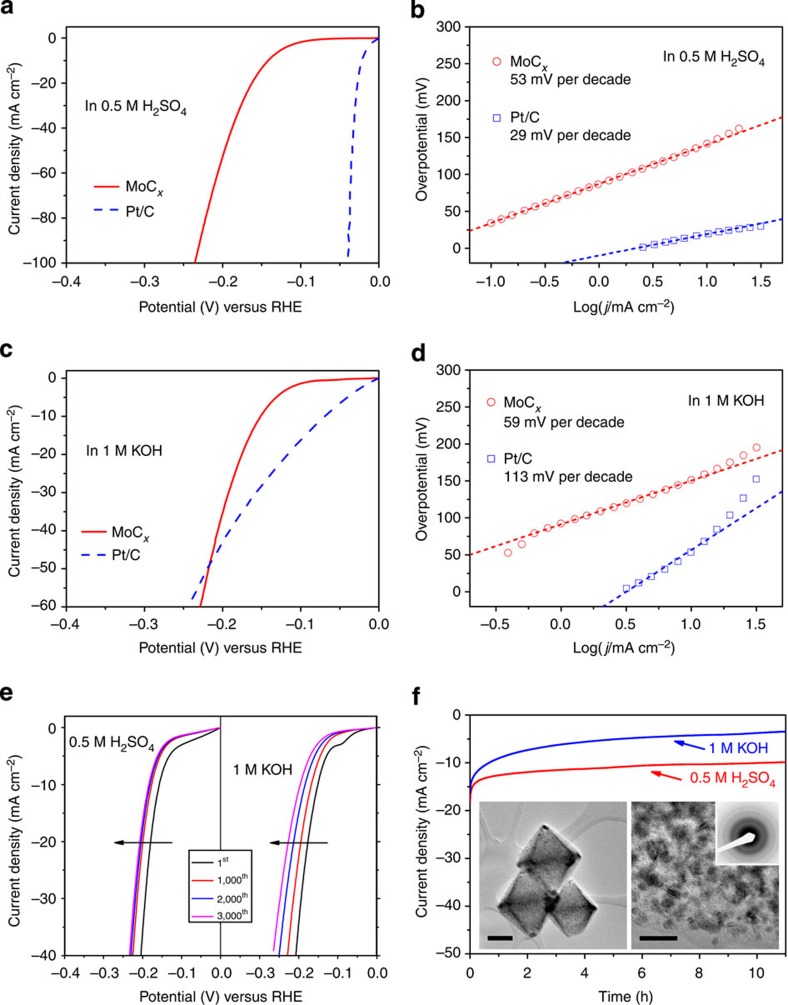
HER performance of porous MoC_*x*_ nano-octahedrons. (**a**) Polarization curve at 2 mV s^−1^ and (**b**) Tafel plots in 0.5 M H_2_SO_4_. (**c**) Polarization curve at 2 mV s^−1^ and (**d**) Tafel plots in 1 M KOH. (**e**) Polarization curves after continuous potential sweeps at 50 mV s^−1^ in 0.5 M H_2_SO_4_ (left) and 1 M KOH (right). (**f**) Time-dependent current density curves under *η*=170 mV in 0.5 M H_2_SO_4_ and *η*=180 mV in 1 M KOH (insets: TEM images and SAED pattern after 5,000 potential sweeps in 0.5 M H_2_SO_4_. Scale bars, left inset (200 nm) and right inset (20 nm)).

The as-prepared porous MoC_*x*_ nano-octahedrons are evaluated as electrocatalysts for HER in both acidic and basic aqueous solutions. The porous MoC_*x*_ nano-octahedrons exhibit optimal performance with a mass loading of 0.8 mg cm^−2^ on a glassy carbon (GC) disk electrode (Supplementary Fig. 7), while the catalysts prepared with shorter carburization time or at higher temperature show slightly inferior performance (Supplementary Fig. 8). The representative polarization curve (current density is based on geometric area of the electrode) and Tafel plot of the MoC_*x*_ electrocatalyst in 0.5 M H_2_SO_4_ are shown in Fig. 4a,b, respectively, along with the performance of the benchmark Pt/C catalyst (40 wt% Pt on carbon black from Johnson Matthey, mass loading of 0.8 mg cm^−2^) for reference. As expected, the Pt/C catalyst exhibits excellent catalytic activity with an onset potential of ~0 V in acidic electrolyte. Meanwhile, the as-prepared MoC_*x*_ electrocatalyst also shows a small onset potential of ~25 mV, estimated from the low current density region of the Tafel plot (Supplementary Fig. 9), beyond which the cathodic current increases rapidly^48^. To achieve current densities (*j*) of 1 and 10 mA cm^−2^, the MoC_*x*_ electrocatalyst requires overpotentials (*η*) of ~87 and 142 mV, respectively. Tafel plots depicted in Fig. 4b show a small Tafel slope of 53 mV per decade for MoC_*x*_ nano-octahedrons, higher than 29 mV per decade for the Pt/C catalyst. By extrapolating the Tafel plot, the exchange current density of MoC_*x*_ nano-octahedrons can be calculated as 0.023 mA cm^−2^. Figure 4c,d shows the electrocatalytic properties of the MoC_*x*_ nano-octahedrons and Pt/C in basic condition. Although the Pt/C catalyst exhibits a small onset potential close to 0 V compared with ~80 mV for the MoC_*x*_ nano-octahedrons (Supplementary Fig. 10), the MoC_*x*_ electrocatalyst outperforms the Pt/C catalyst for *η*≥220 mV with rapidly rising cathodic current. Small overpotentials of 92 and 151 mV are required for the MoC_*x*_ nano-octahedrons to drive *j*=1 and 10 mA cm^−2^, respectively. In addition, the MoC_*x*_ nano-octahedrons exhibit a smaller Tafel slope (59 mV per decade) than the Pt/C catalyst (113 mV per decade) as shown in Fig. 4d, along with an exchange current density of ~0.029 mA cm^−2^. The above comparison is based on the same loading mass of catalysts, which better reflects their performance in practical application and can be directly translated into their relative mass activity. Alternatively, we further compare the current density based on the mass of active materials (MoC for MoC_*x*_ nano-octahedrons and Pt for Pt/C) and the turnover frequency assuming all metal atoms are involved in the HER process (Supplementary Fig. 11). Similar trends are observed. Specifically, the Pt/C catalyst possesses overwhelming advantage in acidic media, while in basic media the activity of MoC_*x*_ nano-octahedrons approaches that of Pt/C catalyst at high overpotential. The electrochemical properties of the MoC_*x*_ nano-octahedrons are summarized in Table 1, demonstrating the remarkable electrocatalytic HER activity in both acidic and basic solutions.

To better understand the origin of such high electrocatalytic performance, we further compare our MoC_*x*_ nano-octahedrons with the irregular MoC_*x*_ nanoparticles (denoted as MoC_*x*_ NPs, as shown in Supplementary Fig. 4 after grinding) as a reference sample, which exhibits similar composition but without well-defined nanostructure. Polarization curves in Supplementary Fig. 12 clearly reveal the much inferior performance of MoC_*x*_ NPs with *η*≈230 mV to drive *j*=1 mA cm^−2^ in both acidic and basic solutions. To reveal whether the high activity comes from increased surface area, we compare the apparent electrochemical surface area (ECSA) of these two electrocatalysts by measuring the double-layer capacitance (*C*_dl_), which is typically used to represent the ECSA (Supplementary Fig. 13)^12^,^18^. Surprisingly, the MoC_*x*_ NPs actually possess quite similar *C*_dl_, equivalent to ECSA, compared with MoC_*x*_ nano-octahedrons. Although the high surface area of MoC_*x*_ nano-octahedrons would obviously result in certain advantages when compared with bulky or low surface area materials, this is not the sole reason accounting for the high electrocatalytic activity.

Electrochemical impedance spectroscopy (EIS) analysis is performed on MoC_*x*_ nano-octahedrons and MoC_*x*_ NPs. Consistent with the previous studies, the EIS Nyquist plots of MoC_*x*_ nano-octahedrons in both acidic and basic solutions exhibit two time constants (Supplementary Fig. 14). The first one at high frequency is related to the surface porosity of the electrode; the second one at low frequency, which depends on the overpotential, reflects the charge transfer process during HER^25^,^27^. Generally speaking, the charge-transfer resistance (*R*_ct_) shows strong correlation with the electrochemical performance. Thus, the Nyquist plots of MoC_*x*_ nano-octahedrons and MoC_*x*_ NPs at given overpotentials (that is, *η*=90 and 190 mV) in 0.5 M H_2_SO_4_ are compared and fitted to an equivalent electrical circuit with two time constants (Supplementary Fig. 15). It can be seen that for both samples the *R*_ct_ substantially reduces at high overpotential. However, the value for MoC_*x*_ nano-octahedrons is much smaller (about one order of magnitude lower) than that for MoC_*x*_ NPs at the same overpotential, in line with their different HER activity. The small charge transfer resistance would be mainly related to the synergistic effect and strong interaction between the MoC_*x*_ nanocrytallites and the continuous and *in situ*-incorporated carbon matrix, which ensures the facile electron transfer in the porous MoC_*x*_ nano-octahedrons. Together with the above analysis of ECSA, we speculate that the high electrochemical activity of MoC_*x*_ nano-octahedrons is due to their improved electronic/chemical properties and/or the exposure of more active sites, which are related to their unique mesoporous structure and small primary nanocrystallites. Nevertheless, more in-depth investigations would be necessary to reveal the detailed mechanism involved.

To assess the durability of the MoC_*x*_ electrocatalyst, accelerated linear potential sweeps are conducted repeatedly on the electrodes at a scan rate of 50 mV s^−1^ as shown in Fig. 4e. In acidic condition, the polarization curves show a small shift of ~25 mV at *j*=10 mA cm^−2^ in the initial 1,000 sweeps, and then appear rather stable afterwards. On the other hand, the MoC_*x*_ nano-octahedrons exhibit a continuous but small loss of activity in basic condition on repeated potential sweeps, implying some minor corrosion of electrocatalyst in the basic electrolyte. It should be noted that loss of electrocatalyst from the electrode on rapid rotation might also account for some degradation of the performance. The stability of the electrocatalyst is also evaluated by prolonged electrolysis at constant potentials, as shown in Fig. 4f. In line with the above studies, the current density of MoC_*x*_ nano-octahedrons generally remains stable in 0.5 M H_2_SO_4_ for more than 10 h, whereas small degradation is observed in 1 M KOH on long-term operation. We have also examined the MoC_*x*_ nano-octahedrons under TEM observation after continuous linear potential sweeps in acidic (insets of Fig. 4f) and basic (Supplementary Fig. 16) media. The nanostructure and crystallinity are well retained after the degradation measurement, again corroborating the good stability in acidic environment. However, some corrosion of the MoC_*x*_ nano-octahedrons (especially the amorphous carbon) occurs during the potential sweeps in 1 M KOH. Such corrosion would cause the disintegration of the MoC_*x*_ nano-octahedrons and loss of active materials, which is expected to account for the small but continuous degradation in basic condition.

## Discussion

The MOFs-assisted strategy presented in this work is facile and easily reproducible to synthesize porous MoC_*x*_ particles composed of a few nanometer-sized nanocrystallites. Compared with other synthesis methods, such as solid–gas phase reaction^49^ and pyrolysis of composites containing metal and carbon sources^24^,^25^, this MOFs-assisted approach guarantees homogeneous and efficient reaction, as well as uniform mesoporosity of the carbide product, originating from the unique crystalline structure with atomically hybridized MOFs matrix and Mo-based POMs. In addition, the highly localized and confined carburization process produces small nanocrystallites, which are embedded in an amorphous carbon matrix and prohibited from excessive growth. More importantly, the present strategy can be easily extended to synthesize tungsten carbide and molybdenum–tungsten mixed carbide (Supplementary Fig. 17), and potentially applicable to other early transition metals as well (Supplementary Note 1).

In virtue of the unique nanostructure, the porous MoC_*x*_ nano-octahedrons exhibit excellent electrocatalytic activity for HER. In acidic aqueous electrolyte, the performance is among the best reported when compared with many representative noble metal-free electrocatalysts, such as various molybdenum-based compounds, transition metal dichalcogenides and phosphides (Supplementary Table 1). The HER performance in basic condition is also compared favourably with many HER catalysts (Supplementary Table 2). In particular, the electrocatalytic activity of MoC_*x*_ nano-octahedrons in acidic media is comparable to the state-of-the-art β-Mo_2_C-based electrocatalysts with extra graphitic carbon supports (for example, graphene and/or carbon nanotubes)^25^,^26^,^50^. However, such high HER activity has not yet been achieved on other phases of molybdenum carbide^41^. Considering the sub-micrometre size of the MoC_*x*_ nano-octahedrons, such catalytic activity is truly impressive. The high HER performance of our MoC_*x*_ nano-octahedrons might be attributed to the following aspects. First, molybdenum carbides possess exceptional intrinsic HER activity, which is probably related to their Pt-like electronic and chemical properties^22^. Second, the small size of primary MoC_*x*_ nanocrystallites and the high porosity render large electrochemical active surface and more importantly, perhaps more active sites to the MoC_*x*_ nano-octahedrons. Third, the uniform morphology and mesoporous structure are expected to facilitate the charge and mass transport within these relatively large octahedral particles, thus promoting the hydrogen production process. Moreover, the amorphous carbon matrix might grant high robustness of the octahedral particles and provide extra protection for the ultrafine MoC_*x*_ nanocrystallites with high surface energy. Nevertheless, the active materials in the centre part of MoC_*x*_ nano-octahedrons might not be fully utilized. Thus, further improvement is highly expected by optimizing the size/porosity of the particles and/or incorporating supports (for example, carbon nanotubes, graphene sheets and integrated current collectors).

In summary, we report a novel MOFs-assisted strategy for synthesizing nanostructured MoC_*x*_ nano-octahedrons as a highly active electrocatalyst for HER. This strategy relies on the confined and *in situ* carburization reaction occurring in a unique MOFs-based compound (NENU-5) consisting of a Cu-based MOFs (HKUST-1) host and guest Mo-based Keggin POMs resided in pores, which enables the uniform formation of metal carbide nanocrystallites without coalescence and excess growth. The as-prepared MoC_*x*_ nano-octahedrons consist of ultrafine nanocrytallites with an unusual *η*-MoC phase embedded in an amorphous carbon matrix, and possess a uniform and highly mesoporous structure. Benefiting from the desirable nanostructure, these porous MoC_*x*_ nano-octahedrons exhibit remarkable electrocatalytic activity for HER in both acidic and basic solutions with good stability. Moreover, such a strategy could be applicable for synthesizing other nanostructured transition metal carbides, thus opening new opportunities to develop high-performance functional materials for various applications.

## Methods

### Synthesis of NENU-5 nano-octahedrons

All chemicals were purchased and used without further purification. In a typical synthesis, 1 mmol of copper (II) acetate monohydrate (Sigma-Aldrich), 0.5 mmol of L-glutamic acid (Sigma-Aldrich) and 0.3 g of phosphomolybdic acid hydrate (Sigma-Aldrich) were dissolved in 40 ml of deionized water and stirred at ambient condition for 20 min. After that, 0.67 mmol of 1,3,5-benzenetricarboxylic acid (Merck) completely dissolved in 40 ml of ethanol was poured into the above solution under continuous stirring. The solution immediately turns turbid due to the rapid formation of NENU-5 nanocrystals. After stirring for 14 h at ambient condition, the green precipitate was collected by centrifugation and washed twice with ethanol. The product was dried at 70 °C overnight for further experiment and characterizations. The size of the NENU-5 particles can be easily tuned by varying the added amount of L-glutamic acid.

### Synthesis of porous MoC_*x*_ nano-octahedrons

The NENU-5 nano-octahedrons were heated in a tube furnace under N_2_ flow with a ramp rate of 2 °C min^−1^, maintained at 800 °C for 6 h and then cooled down naturally. The as-prepared sample was denoted as MoC_*x*_-Cu. The copper particles were removed by dispersing the sample in 0.1 M FeCl_3_ aqueous solution at ambient condition for 2 h. The resulting porous MoC_*x*_ nano-octahedrons were collected by centrifugation, repeatedly washed with deionized water and then dried at 70 °C overnight.

### Materials characterizations

The morphologies and structures of the products were characterized with FESEM (JEOL, JSM-6700 F, 5 kV) and TEM (JEOL, JEM-2010 and JEM-2100F, 200 kV). The chemical compositions and elemental mapping of the samples were analysed by EDX attached to JSM-7600F (FESEM, JEOL, 15 kV) and JEM-2100 F. The crystallographic information was collected by powder XRD (Bruker D8 Advance diffractometer with Cu K*α* radiation *λ*=1.5406 Å). The N_2_ adsorption–desorption isotherms were collected using a Quantachrome Instruments Autosorb AS-6B at liquid-nitrogen temperature. Raman spectrum was collected using a Renishaw system 1000 micro-Raman spectroscope (Renishaw, UK). Thermogravimetric analysis was performed under air flow with a temperature ramp of 10 °C min^−1^.

### Electrochemical measurements

The electrocatalytic activity was evaluated in a three-electrode configuration using a rotating disk electrode (PINE Research Instrumentation, at a rotation speed of 1500, r.p.m.) with an Autolab potentiostat/galvanostat (Model PGSTAT-72637) workstation at ambient temperature. A GC disk electrode (5 mm in diameter) served as the support for the working electrode. The catalyst suspension was prepared by dispersing 10 mg of catalyst in 2 ml of solution containing 1.9 ml of ethanol and 100 μl of 0.5 wt% Nafion solution followed by ultrasonication for 20 min. Then the catalyst suspension was pipetted using a micropipettor on the GC surface. The working electrode was dried at ambient temperature. A saturated calomel electrode (SCE) was used as the reference electrode and a graphite rod was used as the counter electrode. Potentials were referenced to a reversible hydrogen electrode (RHE): E(RHE)=E(SCE)+(0.242+0.059 pH)V. Linear sweep voltammetry was recorded in 0.5 M H_2_SO_4_ (pH=0.3) and 1 M KOH (pH=14) at a scan rate of 2 mV s^−1^ to obtain the polarization curves. The long-term stability tests were performed by continuous linear sweep voltammetry scans from −0.2 to −0.6 V (versus SCE, in 0.5 M H_2_SO_4_) and −1.0 to −1.4 V (versus SCE, in 1 M KOH) at a sweep rate of 50 mV s^−1^. All the data presented were corrected for iR losses and background current. To evaluate the ECSA, cyclic voltammograms were obtained from −0.2 to 0.2 V (versus SCE, in 0.5 M H_2_SO_4_) with sweep rates of 5, 10, 20, 50, 100 mV s^−1^. EIS was performed at various overpotentials with frequency from 0.1 to 100,000 Hz and an amplitude of 10 mV.

## Author contributions

H.B.W. and X.W.L. conceived the idea and co-wrote the manuscript. H.B.W. carried out the synthesis. H.B.W. and B.Y.X. carried out the electrochemical evaluation. L.Y. & X.-Y.Y. helped in materials characterizations.

## Additional information

**How to cite this article**: Wu, H. B. *et al*. Porous molybdenum carbide nano-octahedrons synthesized via confined carburization in metal-organic frameworks for efficient hydrogen production. *Nat. Commun*. 6:6512 doi: 10.1038/ncomms7512 (2015).

## Supplementary Material

Supplementary Information Supplementary Figures 1-17, Supplementary Tables 1-2, Supplementary Note and Supplementary References.

## Figures and Tables

**Table 1:** Summary of HER activities of porous MoC_*x*_ nano-octahedrons.

Electrolyte	Onset *η* (mV versus RHE)	*η* at *j*=1 mA cm^−2^ (mV versus RHE)	*η* at *j*=10 mA cm^−2^ (mV versus RHE)	Tafel slope (mV per decade)	Exchange current density (mA cm^−2^)
0.5 M H_2_SO_4_	~25	87	142	53	0.023
1 M KOH	~80	92	151	59	0.029

HER, hydrogen evolution reaction.
