# Supplementary material for: Porous molybdenum carbide nano-octahedrons synthesized via confined carburization in metal-organic frameworks for efficient hydrogen production
Source: Nat Commun. 2015 Mar 11;6:6512. doi: 10.1038/ncomms7512 (PMC4382699; doi:10.1038/ncomms7512)
Supplement: Supplementary Information — Supplementary Figures 1-17, Supplementary Tables 1-2, Supplementary Note and Supplementary References. [file ncomms7512-s1.pdf]

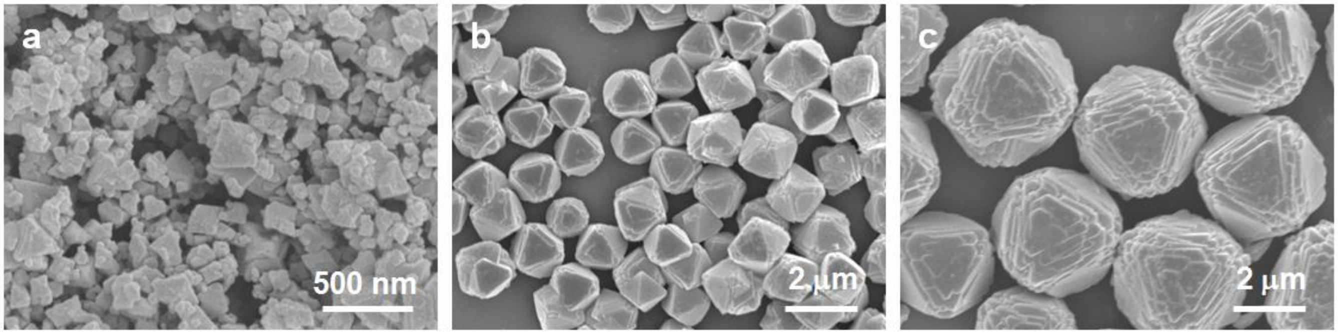

Supplementary Figure 1. FESEM images of NENU-5 particles prepared with different amount of L-glutamic acid: (**a**) 0 mmol, (**b**) 1 mmol, and (**c**) 2 mmol.

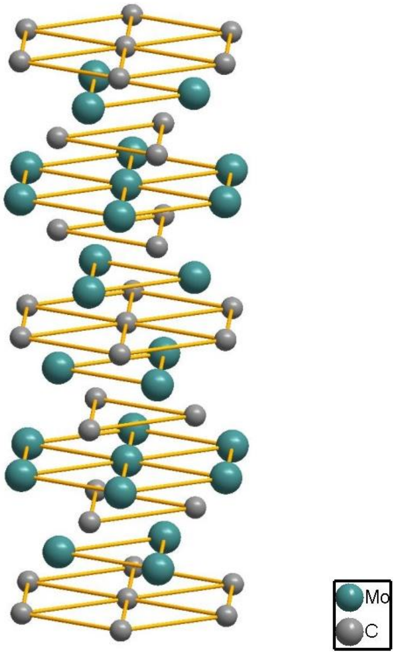

Supplementary Figure 2. A schematic illustration of the crystal structure of stoichiometric η-MoC phase with an ABCACB stacking sequences with planes of Mo and C atoms[1].

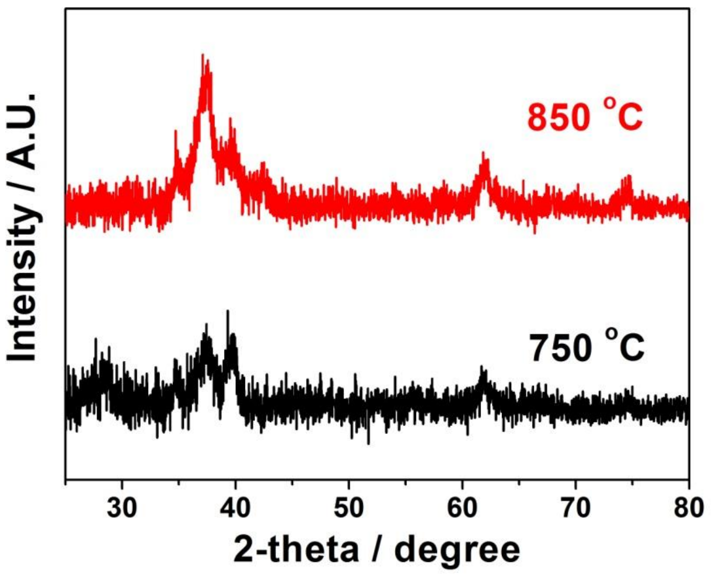

Supplementary Figure 3. XRD patterns of MoC_x prepared at 750 and 850 ^{o}C.

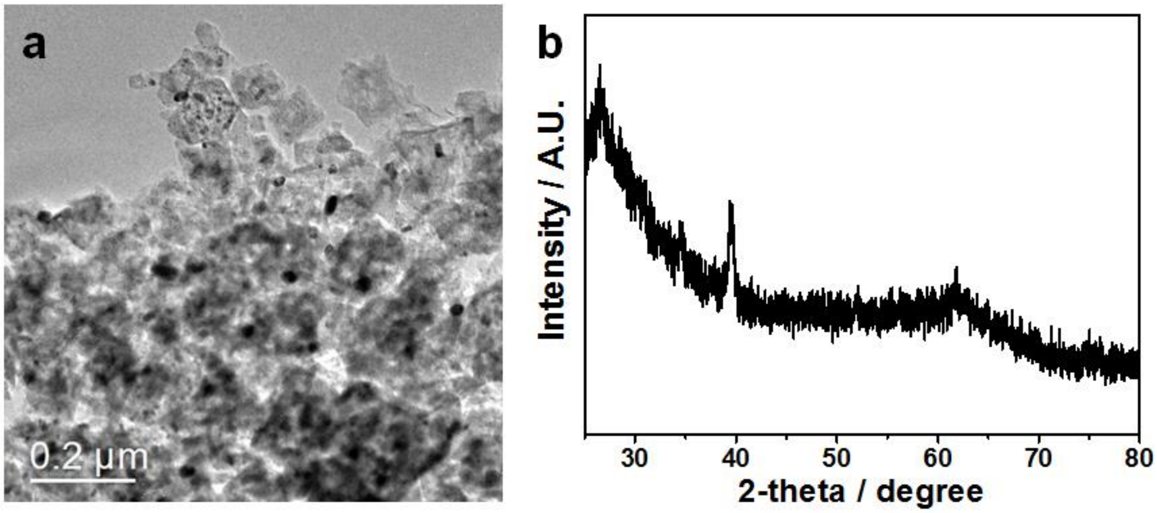

Supplementary Figure 4. (**a**) TEM image and (**b**) XRD pattern of irregular MoC_x nanoparticles prepared using NENU-5 nanoparticles shown in Supplementary Figure 1a.

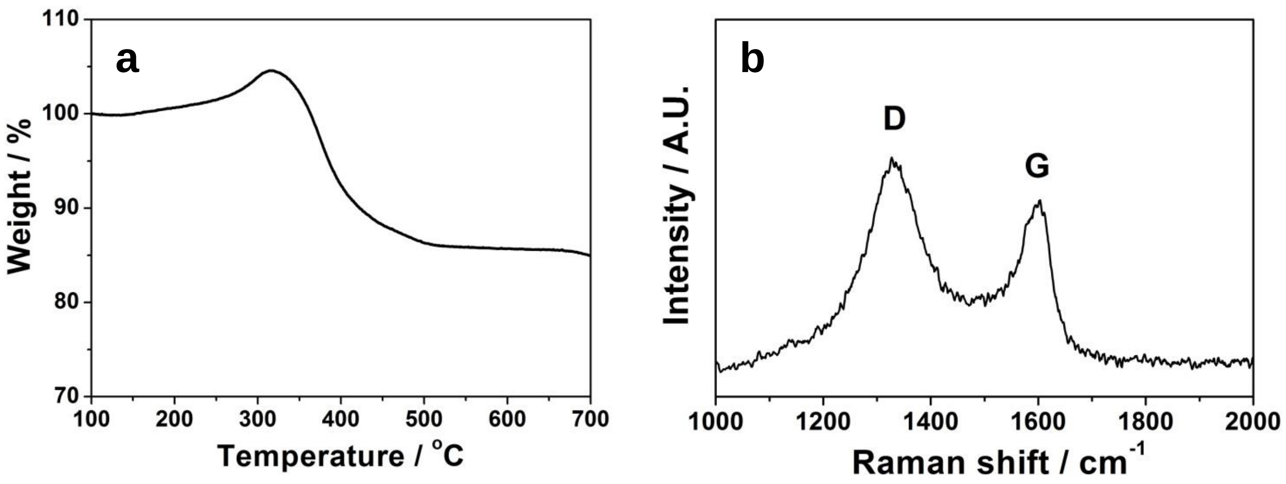

Supplementary Figure 5. (**a**) TGA curve and (**b**) Raman spectrum of MoC_x nano-octahedrons showing the presence of amorphous carbon. For TGA curve, the initial weight gain below 350 ^oC is due to the gradual oxidation of MoC_x to MoO_3, followed by a significant weight loss caused by the combustion of carbon. Assuming that the sample is composed of stoichiometric MoC and carbon, and converts to only MoO_3 after heating to 600 ^oC with remaining weight of ca. 85 wt.%, the carbon content is estimated to be ca. 36 wt.% in the MoC_x nano-octahedrons according to the following equation: m (carbon) = 1 - 85 wt.% * M(MoC)/M(MoO_3) = 1 - 85 wt.% * 108/144 $\approx$ 36 wt.%. For the Raman spectrum, the D band and G band correspond to the disordered graphitic carbon and graphitic carbon, respectively. The lower intensity of G band with respect to D band indicates the amorphous nature of the carbon component.

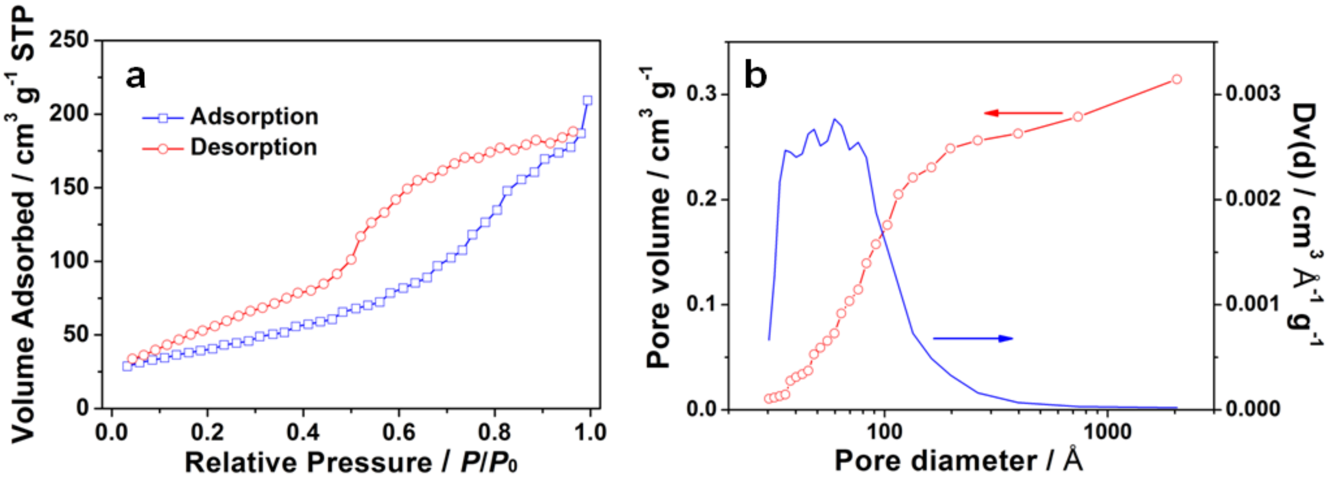

Supplementary Figure 6. (**a**) N_2 adsorption-desorption isotherms and (**b**) pore-size distribution of porous MoC_x nano-octahedrons.

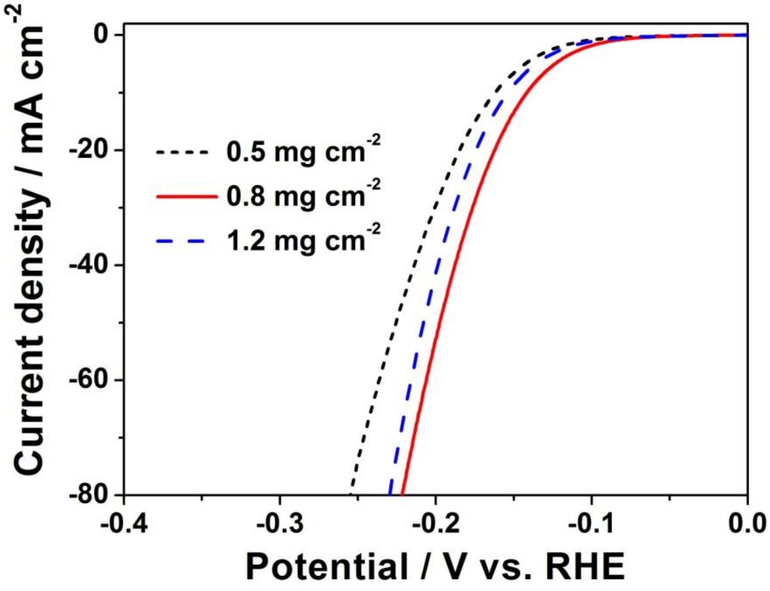

Supplementary Figure 7. Polarization curves of MoC_x nano-octahedrons with different loadings on a glassy carbon electrode in 0.5 M H_2SO_4.

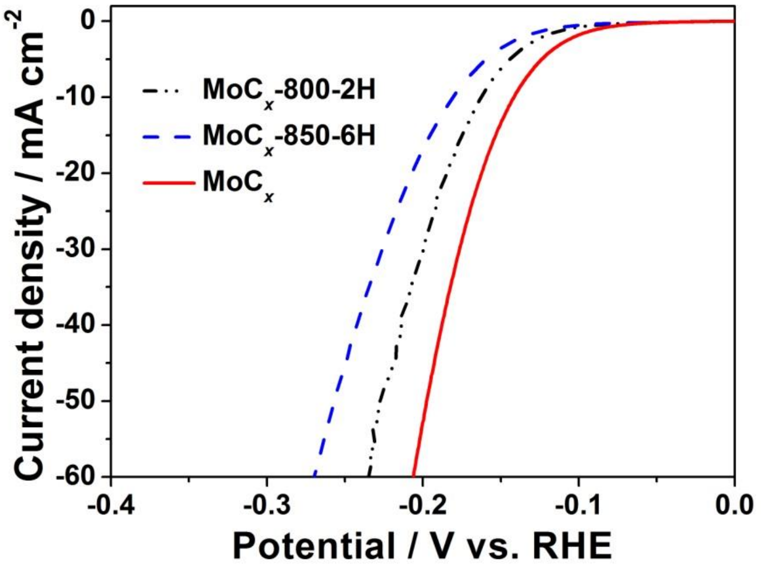

Supplementary Figure 8. Polarization curves of MoC_x nano-octahedrons prepared with shorter reaction time of 2 h (MoC_x-800-2H, black dash dot line), higher reaction temperature of 850 ^{o}C (MoC_x-850-6H, blue dash line), and the main MoC_x sample prepared at 800 ^{o}C for 6 h (MoC_x, red solid line) in 0.5 M H_2SO_4. Both the MoC_x-800-2H and MoC_x-850-6H samples exhibit slightly inferior hydrogen production activity, which is probably due to either the insufficient crystallinity with short reaction time or the aggregation of MoC_x nanocrystallites at high temperature.

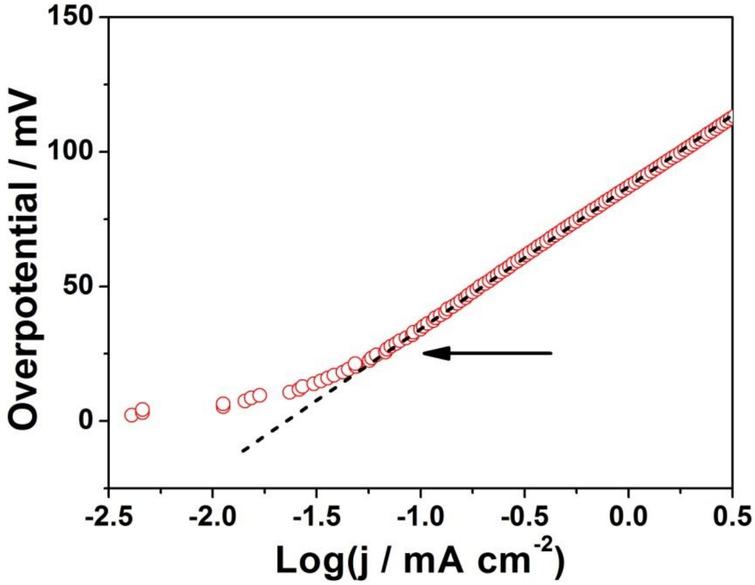

Supplementary Figure 9. Tafel plot in the region of low current densities of MoC_x nano-octahedrons in 0.5 M H_2SO_4. The onset overpotential is determined by the potential when the plot starts to deviate from the linear region as indicated by the arrow.

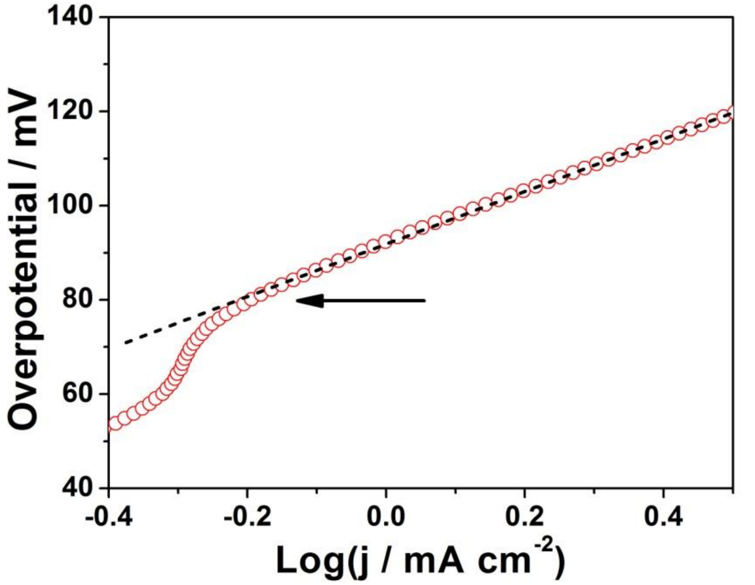

Supplementary Figure 10. Tafel plot in the region of low current densities of MoC_x nano-octahedrons in 1 M KOH.

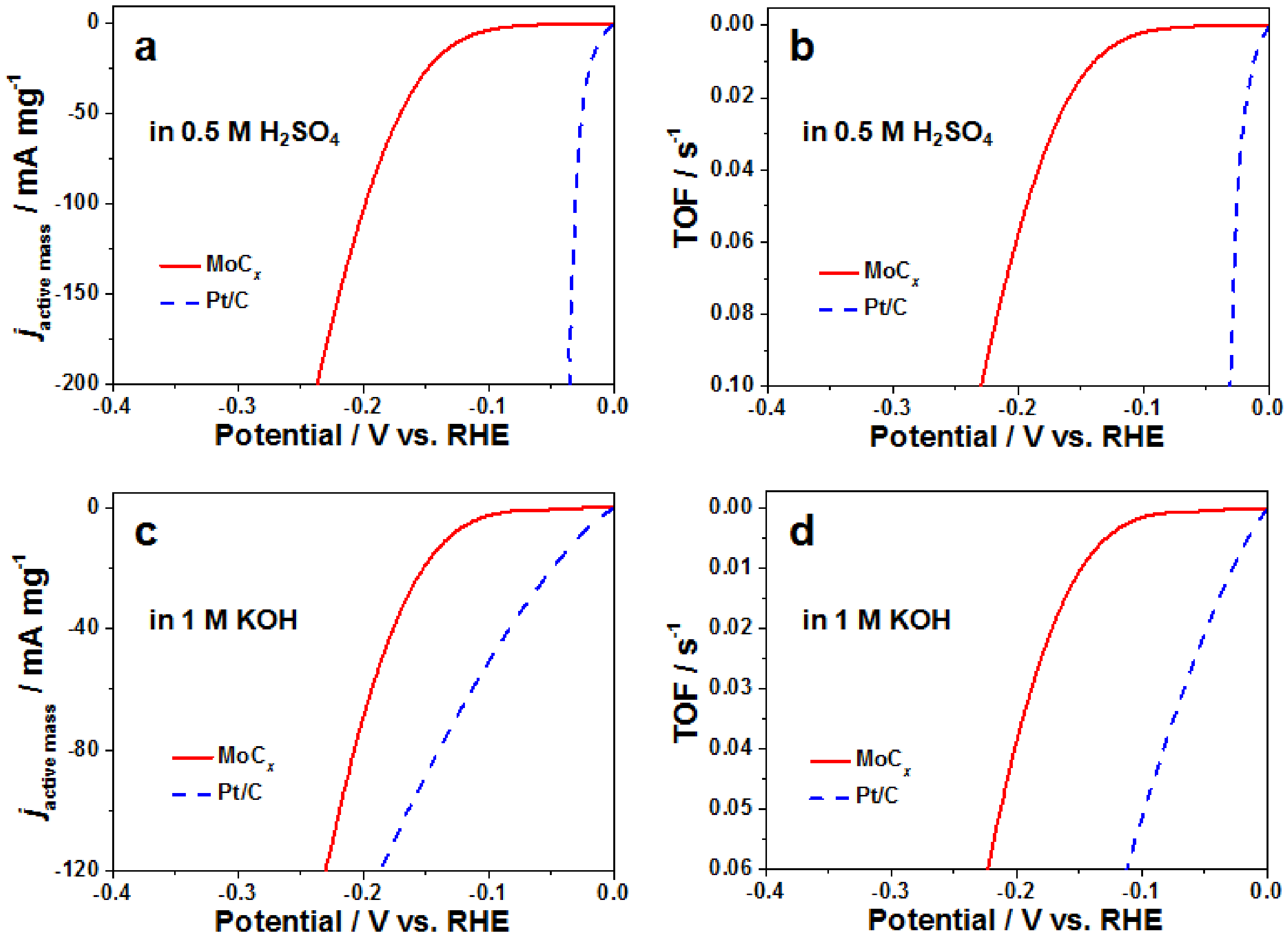

Supplementary Figure 11. Plots of (**a**, **c**) mass activity based on the mass of MoC (64 wt.%) for MoC_x nano-octahedrons and Pt (40 wt.%) for Pt/C and (**b**, **d**) turnover frequency (TOF) assuming all metal atoms are involved *vs.* potential in (**a**, **b**) acidic and (**c**, **d**) basic media. Calculation methods: $j_{\text{active mass}} = j/m$, where j is the current density and m is the loading mass of MoC or Pt on the electrode; TOF = jM/2Fm, where j is the current density, F is Faraday's constant (96485.3 C mol^{-1}), M is the molar mass of MoC or Pt, m is the loading mass of MoC or Pt on the electrode, and number 2 means 2 electrons per mole of H_2. The calculation method used here substantially underestimates the real TOFs of the catalysts, especially for MoC_x nano-octahedrons due to the large particle size.

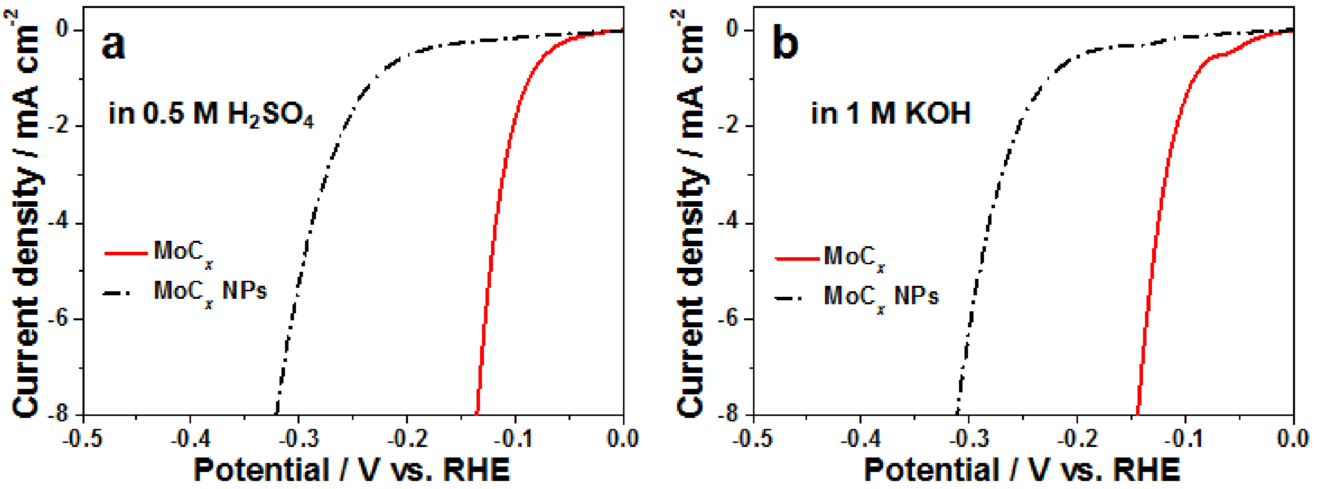

Supplementary Figure 12. Polarization curves of MoC_x nano-octahedrons (MoC_x, red solid lines) and MoC_x nanoparticles (MoC_x NPs, black dash dot lines) in (**a**) acidic and (**b**) basic media.

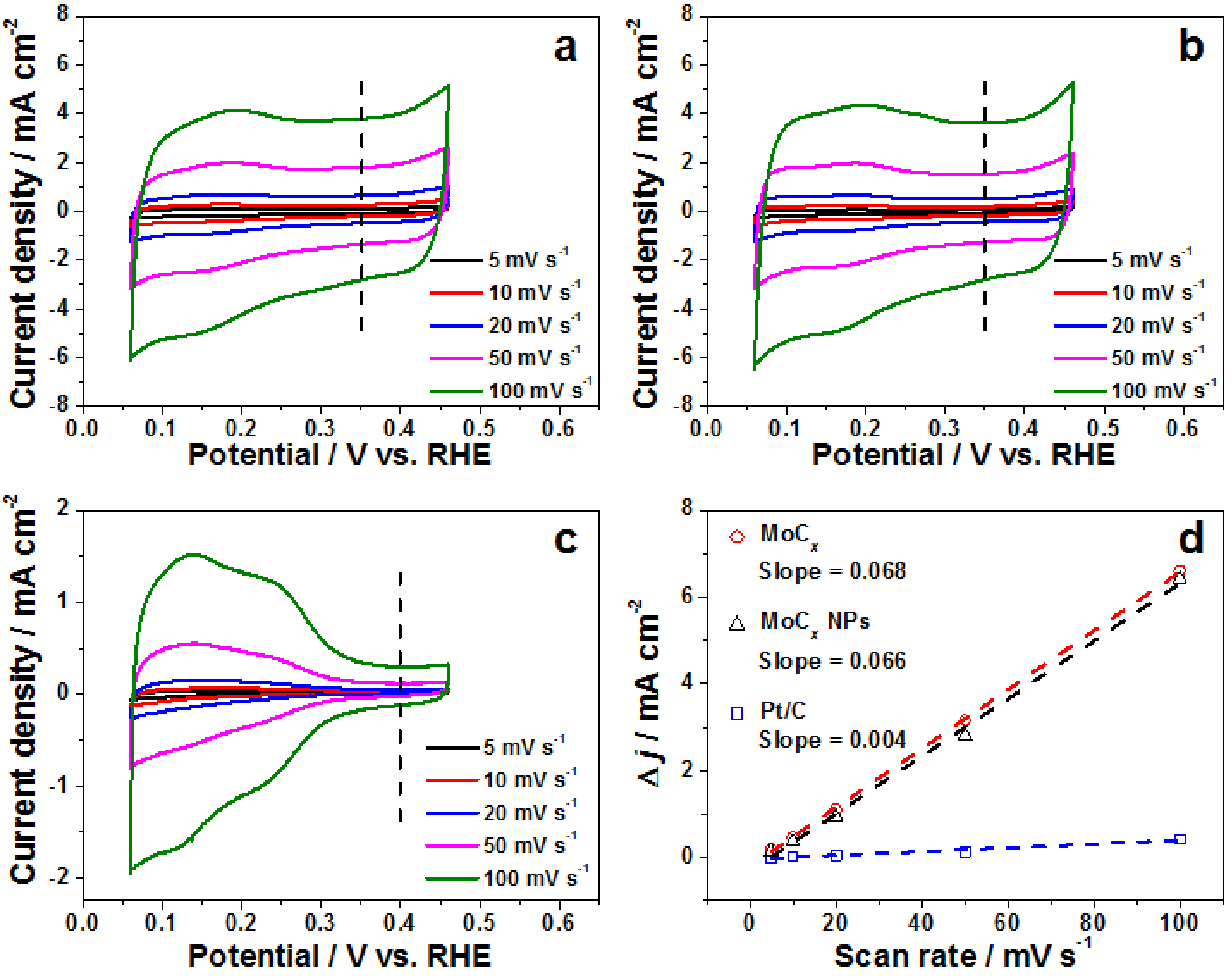

Supplementary Figure 13. Cyclic voltammograms (CVs) of (**a**) MoC_x nano-octahedrons, (**b**) MoC_x NPs, and (**c**) Pt/C in 0.5 M H_2SO_4. (**d**) Current density differences (Δj) plotted against scan rates. Δj is the difference between anodic and cathodic current densities at potential indicated by the black dash lines, where no redox current peaks are observed. The linear slopes in (**d**) are equivalent to twice of the electrochemical double-layer capacitances (C_{dl}). C_{dl} is used to represent and compare the apparent electrochemical surface area (ECSA) between MoC_x nano-octahedrons and MoC_x NPs because of their similar composition, although the contribution from carbon and MoC_x cannot be differentiated. The C_{dl} of Pt/C is also estimated and compared using the same method. However, it is not very accurate to compare the ECSA of Pt/C in term of its C_{dl} due to the different composition and carbon content. Thus, the data of Pt/C in the graphs are only provided for a very rough comparison.

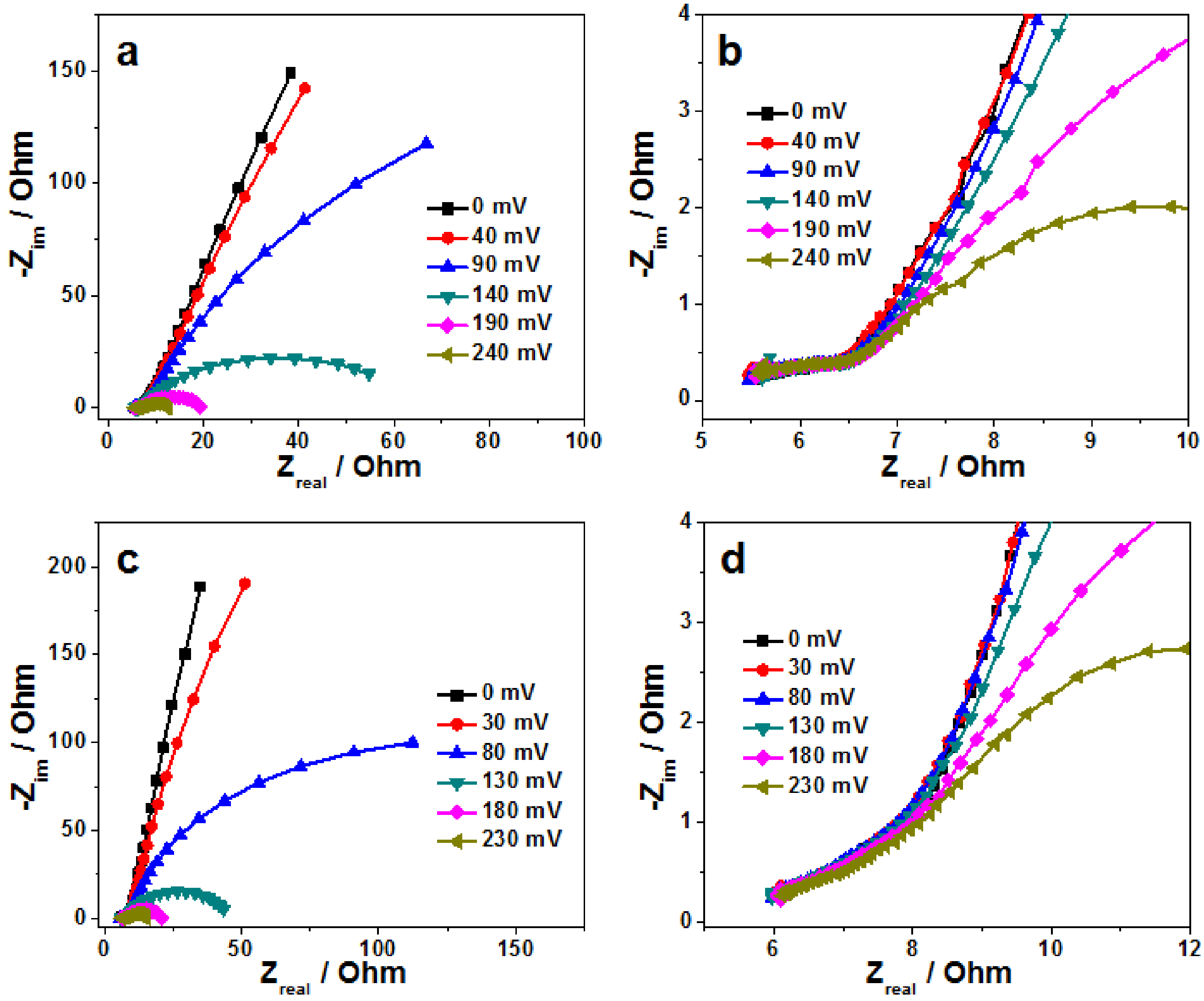

Supplementary Figure 14. Electrochemical impedance spectroscopy (EIS) Nyquist plots of MoC_x nano-octahedrons in (**a**, **b**) 0.5 M H_2SO_4 and (**c**, **d**) 1 M KOH at various overpotentials. (**b**) and (**d**) show the high frequency regions of (**a**) and (**c**), respectively.

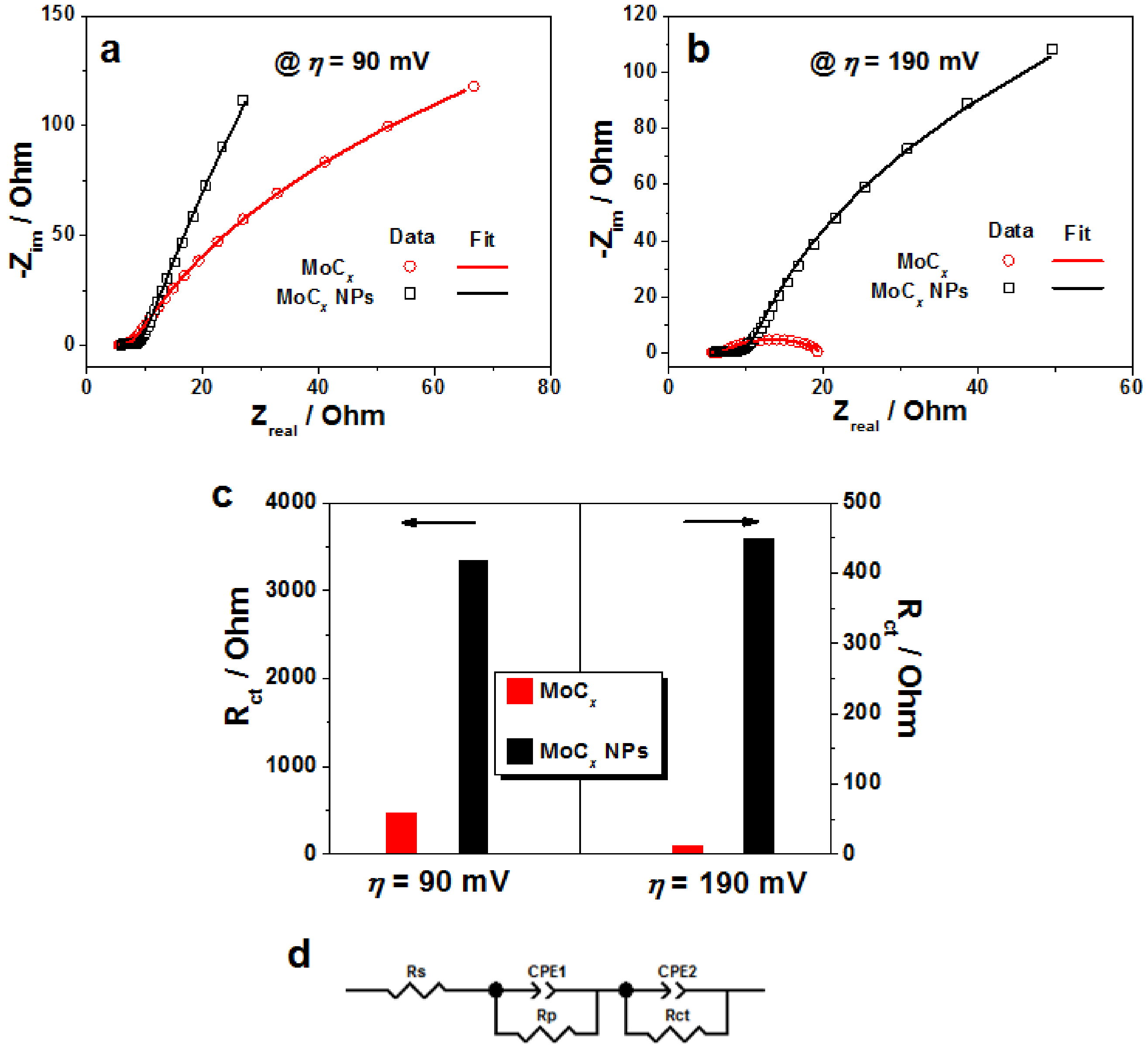

Supplementary Figure 15. Comparison and fitting of Nyquist plots of MoC_x nano-octahedrons and MoC_x NPs in 0.5 M H_2SO_4 at (**a**) η = 90 mV and (**b**) η = 190 mV. (**c**) Comparison of charge transfer resistance (R_{ct}) at η = 90 mV (left) and η = 190 mV (right). (**d**) Equivalent electrical circuit used to model the EIS data, consisting of a series resistance (R_s), two constant phase elements (CPE1 and CPE2), resistance related to surface porosity (R_p), and charge transfer resistance related to HER process (R_{ct}).

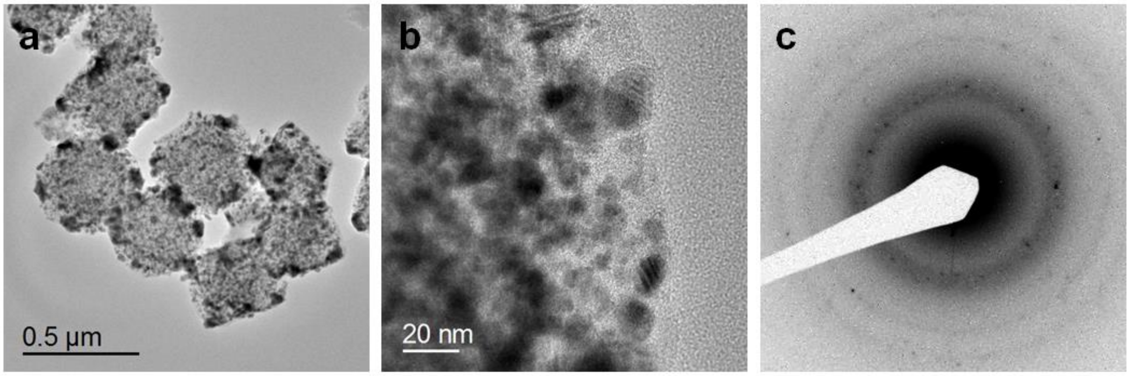

Supplementary Figure 16. (**a**, **b**) TEM images and (**c**) SAED pattern of MoC_x nano-octahedrons after 3000 potential sweeps in 1 M KOH. The shape of the MoC_x nano-octahedrons becomes less well-defined. A closer observation in (**b**) shows that the amorphous carbon layer appears to be corroded and becomes thinner, probably resulting in the isolation and loss of MoC_x nanocrystallites.

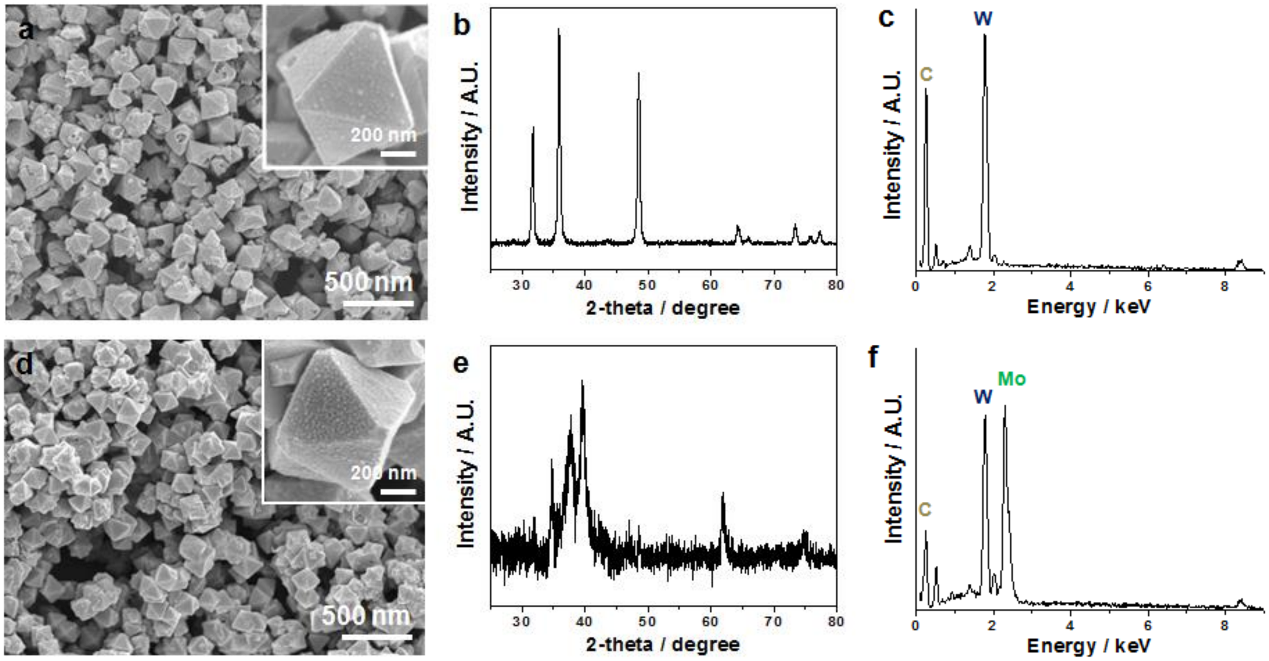

Supplementary Figure 17. (**a**, **d**) FESEM images, (**b**, **e**) XRD patterns, and (**c**, **f**) EDX spectra of (**a** - **c**) tungsten carbide and (**d** - **f**) molybdenum-tungsten carbide. The precursor for tungsten carbide was prepared with 0.5 g of phosphotungstic acid hydrate (Sigma-Aldrich) and 2 mmol of L-glutamic acid. The precursor for molybdenum-tungsten carbide was prepared with 0.15 g of phosphomolybdic acid hydrate and 0.25 g of phosphotungstic acid hydrate, and 1 mmol of L-glutamic acid. The annealing temperature was 900 ^{o}C for both samples. Other conditions remain unchanged.

Supplementary Table 1. Comparison of HER performance in acid media for MoC_x nano-octahedrons with other electrocatalysts.[a]

Catalysts	Loading mass (mg cm^{-2})	Current density (j, mA cm^{-2})	η at corresponding j (mV)	Tafel slope (mV $decade^{-1}$)
MoC_x (this work)	0.8	1	87	53
		10	142	
Mo_2C and MoB microparticles[2]	1.4-2.5	20	~225[b]	55-56
Double-gyroid MoS_2[3]	-	2	190	50
Aligned MoS_2 film[4]	0.022	6.74	200	43-47
Amorphous MoS_x[5]	0.013-0.282	10	160-235	39-63
$NiMoN_x$ nanosheets[6]	0.25	2	170	35.9
WS_2 nanosheets[7]	≤0.0065	10	~240[b]	55
$WS_{2(1-x)}Se_{2x}$ nanotubes[8]	0.21	10	~260[b]	105
Pyrite-type transition metal dichalcogenides[9]	-	4	190-250[b]	40-70
Ni_2P nanoparticles[10]	1	20	130	46
FeP nanosheets[11]	0.28	10	~240[b]	67
Cu_3P nanowrie arrays[12]	15.2	1	79	67
		10	143	
Mo_2C/CNT[13]	2	10	152	65
Mo_2C/CNT-graphene[14]	0.65-0.67	10	130	58
Mo_2C/RGO[15]	0.285	10	130	57.3

[a] The measurement method of onset overpotential might vary from different reports. Therefore, the overpotential required for driving a particular current density is used to assess the performance in a more practical circumstance.

[b] Estimated from the polarization curves.

Supplementary Table 2. Comparison of HER performance in basic media for MoC_x nano-octahedrons with other electrocatalysts.

Catalysts	Loading mass (mg cm^{-2})	Current density (j, mA cm^{-2})	η at corresponding j (mV)	Tafel slope (mV decade^{-1})
MoC_x (this work)	0.8	1	92	59
		10	151	
Mo_2C and MoB microparticles[2]	0.8-2.3	20	210-240[a]	54-59
Ni-Mo nanopowder[16]	1	10	~80[a]	-
NiO/Ni-CNT[17]	0.4	10	80	82
Co-embedded N-rich CNTs[18]	0.28	1	160	-
		10	370	
Co@Co-oxo/hydroxo phosphate[19]	-	2	385	140
CoP nanowire arrays[20]	0.92	1	115	129
		10	209	

[a] Estimated from the polarization curves.

Supplementary Note 1: POMs based on various early transition metals (e.g., Mo, W, V, Nb) have been reported[21]. In principle, other early transition metal carbides (VC_x, NbC_x, and mixed metal carbides, etc.) can also be synthesized via a similar route using proper POMs-contained MOFs. In this work, Mo/W-based carbides are chosen as representatives to demonstrate the feasibility of the as-proposed MOFs-assisted strategy mainly due to the commercial availability of Mo/W-based POMs, easy incorporation of them into HKUST-1, and the extensive study of Mo/W carbides in catalysis. Moreover, the as-proposed MOFs-assisted confined carburization strategy is not limited to POMs-containing MOFs. It is highly possible to infiltrate other early transition metal-containing precursors such as salts and alkoxides into pre-synthesized MOFs, and carry out similar confined carburization reaction. Thus, the versatility and generality of this MOFs-assisted strategy would be significantly broadened.

Supplementary References

1. Hugosson, H. W. *et al.* Theory of phase stabilities and bonding mechanisms in stoichiometric and substoichiometric molybdenum carbide. *J. Appl. Phys.* **86**, 3758-3767 (1999).
2. Vrubel, H. & Hu, X. Molybdenum Boride and Carbide Catalyze Hydrogen Evolution in both Acidic and Basic Solutions. *Angew. Chem. Int. Ed.* **51**, 12703-12706 (2012).
3. Kibsgaard, J., Chen, Z., Reinecke, B. N. & Jaramillo, T. F. Engineering the surface structure of MoS_2 to preferentially expose active edge sites for electrocatalysis. *Nat. Mater.* **11**, 963-969 (2012).
4. Wang, H. *et al.* Electrochemical tuning of vertically aligned MoS_2 nanofilms and its application in improving hydrogen evolution reaction. *Proc.. Natl. Acad. Sci.* **110**, 19701-19706 (2013).
5. Morales-Guio, C. G. & Hu, X. Amorphous Molybdenum Sulfides as Hydrogen Evolution Catalysts. *Acc. Chem. Res.* **47**, 2671-2681 (2014).
6. Chen, W.-F. *et al.* Hydrogen-Evolution Catalysts Based on Non-Noble Metal Nickel–Molybdenum Nitride Nanosheets. *Angew. Chem. Int. Ed.* **51**, 6131-6135 (2012).
7. Voiry, D. *et al.* Enhanced catalytic activity in strained chemically exfoliated WS_2 nanosheets for hydrogen evolution. *Nat. Mater.* **12**, 850-855 (2013).
8. Xu, K. *et al.* Component-Controllable $WS_{2(1-x)}Se_{2x}$ Nanotubes for Efficient Hydrogen Evolution Reaction. *ACS Nano* **8**, 8468-8476 (2014).
9. Kong, D., Cha, J. J., Wang, H., Lee, H. R. & Cui, Y. First-row transition metal dichalcogenide catalysts for hydrogen evolution reaction. *Energy Environ. Sci.* **6**, 3553-3558 (2013).

10. Popczun, E. J. *et al.* Nanostructured Nickel Phosphide as an Electrocatalyst for the Hydrogen Evolution Reaction. *J. Am. Chem. Soc.* **135**, 9267-9270 (2013).

11. Xu, Y., Wu, R., Zhang, J., Shi, Y. & Zhang, B. Anion-exchange synthesis of nanoporous FeP nanosheets as electrocatalysts for hydrogen evolution reaction. *Chem. Commun.* **49**, 6656-6658 (2013).

12. Tian, J., Liu, Q., Cheng, N., Asiri, A. M. & Sun, X. Self-Supported Cu_3P Nanowire Arrays as an Integrated High-Performance Three-Dimensional Cathode for Generating Hydrogen from Water. *Angew. Chem. Int. Ed.* **53**, 9577-9581 (2014).

13. Chen, W. F. *et al.* Highly active and durable nanostructured molybdenum carbide electrocatalysts for hydrogen production. *Energy Environ. Sci.* **6**, 943-951 (2013).

14. Youn, D. H. *et al.* Highly Active and Stable Hydrogen Evolution Electrocatalysts Based on Molybdenum Compounds on Carbon Nanotube–Graphene Hybrid Support. *ACS Nano* **8**, 5164-5173 (2014).

15. Pan, L. F. *et al.* Molybdenum carbide stabilized on graphene with high electrocatalytic activity for hydrogen evolution reaction. *Chem. Commun.* **50**, 13135-13137 (2014).

16. McKone, J. R., Sadtler, B. F., Werlang, C. A., Lewis, N. S. & Gray, H. B. Ni–Mo Nanopowders for Efficient Electrochemical Hydrogen Evolution. *ACS Catal.* **3**, 166-169 (2012).

17. Gong, M. *et al.* Nanoscale nickel oxide/nickel heterostructures for active hydrogen evolution electrocatalysis. *Nat. Commun.* **5**, 4695 (2014).

18. Zou, X. *et al.* Cobalt-Embedded Nitrogen-Rich Carbon Nanotubes Efficiently Catalyze Hydrogen Evolution Reaction at All pH Values. *Angew. Chem. Int. Ed.* **53**, 4372-4376 (2014).

19. Cobo, S. *et al.* A Janus cobalt-based catalytic material for electro-splitting of water. *Nat. Mater.* **11**, 802-807 (2012).

20. Tian, J., Liu, Q., Asiri, A. M. & Sun, X. Self-Supported Nanoporous Cobalt Phosphide Nanowire Arrays: An Efficient 3D Hydrogen-Evolving Cathode over the Wide Range of pH 0–14. *J. Am. Chem. Soc.* **136**, 7587-7590 (2014).

21. Long, D.-L., Tsunashima, R. & Cronin, L. Polyoxometalates: Building Blocks for Functional Nanoscale Systems. *Angew. Chem. Int. Ed.* **49**, 1736-1758 (2010).
